# Association between Serum Uric Acid Levels and Bone Mineral Density in Taiwanese Elderly Population

**DOI:** 10.3390/ijerph20043448

**Published:** 2023-02-16

**Authors:** Pei-Ting Chung, Hsiao-Chi Ma, Sin-Yi Huang, Hsin-Ning Lien, Kuan-Hsun Ho, Hsin-Yin Hsu, Lee-Ching Hwang, Meng-Ting Tsou, Hsin-Hui Lin, Hsin-Lung Chan

**Affiliations:** 1 Department of Family Medicine, Mackay Memorial Hospital, Taipei 104217, Taiwan; 2 Department of Family Medicine, Mackay Memorial Hospital, Tamsui Branch, New Taipei City 251020, Taiwan; 3 Institute of Epidemiology and Preventive Medicine, College of Public Health, National Taiwan University, Taipei 100025, Taiwan; 4 Department of Medicine, Mackay Medical College, New Taipei City 252005, Taiwan; 5 Mackay Junior College of Medicine, Nursing and Management, New Taipei City 252005, Taiwan

**Keywords:** osteoporosis, osteopenia, bone mineral density, hyperuricemia

## Abstract

Osteoporosis is a progressive metabolic bone disorder characterised by a decline in bone mineral density (BMD). Some previous studies have reported a controversial relationship between uric acid and osteoporosis. This cross-sectional study aimed to assess the association between serum uric acid levels and BMD in older adults from Taiwan. Data from participants aged ≥ 60 years were collected from 2008 to 2018. Furthermore, the participants were classified based on uric acid level quartiles. Regression models were used to assess the association between uric acid levels and bone health, including BMD values and risk of at least osteopenia. Crude and adjusted models of potential confounders, including age, sex and body mass index (BMI), were used. Compared with the first quartile of uric acid levels, the odds ratios for osteoporosis decreased in the higher uric acid level groups after adjustment for age, sex and BMI. The boxplot analysis showed that BMD values were higher in the groups with higher uric acid levels; moreover, the results of the multivariable linear regression model showed a consistent pattern. Notably, uric acid levels were positively correlated with BMD values. Higher uric acid levels in the elderly population might decrease the risk of at least osteopenia. As opposed to the anti-hyperuricemic policy for younger adults with a relatively lower risk of osteoporosis, BMD evaluation and urate-lowering therapy, goal adjustments should be considered for older adults with lower uric acid levels.

## 1. Introduction

Osteoporosis and osteopenia are conditions characterised by a decline in bone mineral density (BMD) [1]. Fragile skeletal microarchitecture increases the danger of a fracture, especially in elderly individuals and post-menopausal women, and may lead to immobilisation and a lower quality of life.

Hyperuricemia is defined as high levels of blood uric acid. The condition is commonly caused by urate overproduction and decreased uric acid excretion. Although hyperuricemia is a crucial etiologic factor of gout, it is also associated with cardiovascular and neurological disease, because uric acid participates in the inflammatory response [2].

Although hyperuricemia is linked with the systemic diseases noted above, uric acid also has some benefits [3,4]. There is growing evidence supporting the protective effect of higher uric acid on the constant production and degradation of bone tissue. Recent studies have shown a positive correlation between uric acid and bone mass among most adults, including post-menopausal women and the elderly [5,6,7,8]. The probable mechanism involves uric acid acting as a powerful antioxidant against free radicals, which cause local tissue damage to bone [9]. Consequently, increased uric acid decreases oxidative stress and prevents the loss of skeletal system mass. However, some studies have not supported the beneficial effect of uric acid on bone metabolism [10,11]. Therefore, the influence of uric acid remains controversial.

Taiwan has become an aged society, and the medical needs of the elderly population are receiving increasing attention. Urate-lowering therapy was prescribed to hyperuricemia patients, but could older adults benefit as much as younger adults? This research aimed at investigating the relationship between uric acid and BMD in a Taiwanese elderly population (age ≥ 60 years). In our research, we estimated the odds ratios of osteoporosis and osteopenia in participants with hyperuricemia compared with controls.

## 2. Materials and Methods

### 2.1. Study Population

We included subjects aged ≥ 60 years who were identified through health examinations at a medical centre in Northern Taiwan to evaluate the association between hyperuricemia and BMD. The exclusion criteria were the presence of systemic inflammatory rheumatic diseases, hematologic diseases, chronic respiratory diseases, gastrointestinal diseases and systemic or localised infectious diseases. A total of 2250 participants underwent health examinations between 2008 and 2018 for this cross-sectional study. The study protocol was evaluated and approved by the Human Research Ethics Committee of Mackay Memorial Hospital (project research number 18MMHIS137).

### 2.2. Data Collection

To obtain their medical history, all of the participants were requested to complete a detailed questionnaire, which consisted of personal health conditions, current medical treatment and demographic characteristics. We also collected data on the body mass index (BMI), blood pressure (mmHg) and waist circumference (cm), as well as other biomarker measurements that included complete blood counts, glucose (mg/dL), lipid profiles, kidney function tests, liver function tests and uric acid levels (mg/dL). The BMI was calculated by dividing the body weight (kg) by the square of the participant’s height (m^2^).

In the statistical analysis, the participants were categorised into four groups based on quartiles of uric acid levels: first quartile (Q1): <4.7 mg/dL, second quartile (Q2): 4.7–5.6 mg/dL, third quartile (Q3): 5.7–6.5 mg/dL and fourth quartile (Q4): ≥6.6 mg/dL. We also established a subgroup with uric acid levels >6.6 mg/dL. In the subgroup, the subjects were categorised on the basis of different uric acid levels, such as Q1: <6.6 mg/dL, Q2: 6.6–7.5 mg/dL, Q3: 7.6–8.5 mg/dL and Q4: ≥8.5 mg/dL.

We measured the BMD in each subject at the lumbar spine and left total hip via dual-energy X-ray absorptiometry (Lunar Prodigy Advance; GE Healthcare, Madison, WI, USA). According to the World Health Organization, we defined osteoporosis as BMD values that are more than 2.5 standard deviations (SDs) below the mean value for general young adults (T score ≤ −2.5). As proposed by the International Society for Clinical Densitometry, this definition is based on the lowest T score of the measured skeletal site. Furthermore, osteopenia was defined as a T score between 1 and 2.5 SDs below the general young adult mean (−1.0 > T score ≥ −2.5). We also defined at least osteopenia (T score ≤ −1.0) by the composite of osteoporosis and osteopenia.

### 2.3. Statistical Analysis

All statistical data were analysed using SPSS version 20.0 software (IBM SPSS Statistics for Windows, IBM Corp., Armonk, NY, USA). Data are demonstrated as percentages and frequencies for categorical data. We used the chi-square test to analyse the categorical variables. An independent *t*-test was performed to compare discrete variables.

Regression models were used to evaluate the association between the uric acid level and bone health, including BMD values and the risk of at least osteopenia (the composite of osteoporosis and osteopenia). Adjusted models of potential confounding factors, including age, sex and BMI, were performed by a multivariate logistic regression analysis. Model 1 was adjusted by stratification of the age groups (60–69 years, 70–79 years and ≥80 years). In model 2, we added the sex and BMI groups (<24 kg/m^2^, 24–26.9 kg/m^2^ and ≥27 kg/m^2^) as potential confounders.

To describe the relationship between different uric acid levels and at least osteopenia, we calculated the odds ratio (OR) and 95% confidence interval (CI). The hip and lumbar spine BMD trends for the different serum uric acid levels were evaluated using the *p*-values from the trend test. A *p*-value of <0.05 was considered to be statistically significant.

## 3. Results

The 2250 participants’ ages ranged from 60 to 89 years, with an average of 65.9 ± 5.5 years. According to the serum uric acid levels, all participants were divided into the following groups, as presented in Table 1: <4.7 mg/dL, 4.7–5.6 mg/dL, 5.7–6.5 mg/dL and ≥6.6 mg/dL. There were significant differences in the general characteristics between the serum uric acid level groups, with the exception of the diagnosis of diabetes mellitus and thyroid disease. Compared with the other groups, the participants with the highest serum uric acid level were more likely to be men (*p* < 0.001) and aged ≥80 years (*p* < 0.05) and more likely to suffer from hypertension (*p* < 0.05), hyperlipidaemia (*p* < 0.001) and chronic kidney disease (CKD, *p* < 0.001), which was defined as eGFR < 60 mL/min/1.73 m^2^. These participants also had a higher BMI (*p* < 0.001). Overall, they were less likely to have osteopenia (*p* < 0.001).

Table 2 shows the results of the multiple logistic regression analysis for OR of the serum uric acid categories for at least osteopenia. In the unadjusted model, participants in the highest uric acid level group were 0.38 times less likely to have osteopenia than those in the lowest serum uric acid level group (95% CI: 0.29–0.50, *p* < 0.001). After adjustment for confounders, this positive association still existed in model 1 (OR, 0.54, 95% CI: 0.41–0.73, *p* < 0.001) and model 2. Compared with the lowest uric acid level group, the OR for at least osteopenia was 0.91 (95% CI: 0.68–1.22, *p* = 0.54) for the second quartile uric acid level, 0.89 (0.66–1.20, *p* = 0.44) for the third quartile uric acid level and 0.68 (0.50–0.92, *p* < 0.05) for the highest uric acid level group (*p* for the trend was <0.001) after adjustment for age, sex and BMI.

**Figure 1:**
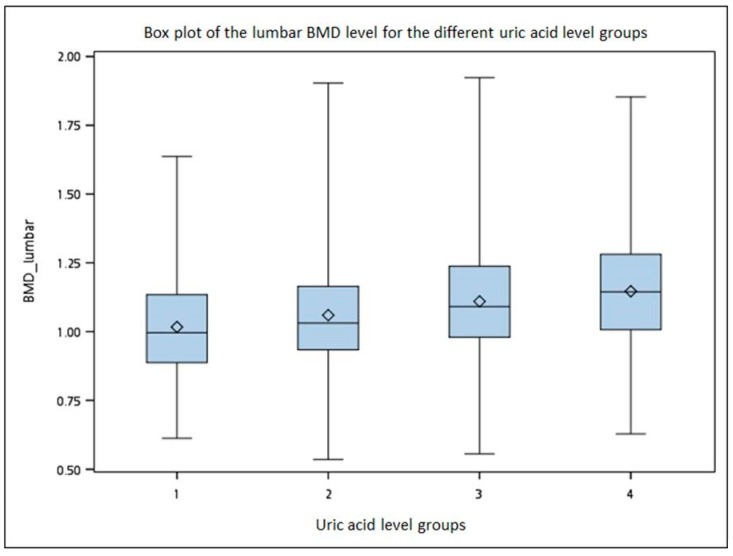
Boxplot showing the bone mineral density (BMD) level of each group based on their uric acid levels.

Figure 1 shows a boxplot of the BMD level for the different uric acid level groups, which demonstrated that a higher BMD was noted in the higher uric acid level group.

The association between serum uric acid and BMD from the multivariable linear regression model is presented in Table 3. The serum uric acid level was positively correlated with both lumbar BMD (*β* = 0.011, *p* < 0.05) and hip BMD (*β* = 0.005, *p* < 0.05).

As shown in Table 4, we focused on the participants with hyperuricemia. In the unadjusted model, participants in the highest uric acid level group were 0.44 times less likely to have at least osteopenia than those in the normal serum uric acid level group (95% CI: 0.26–0.74, *p* < 0.001). After adjustment for confounders, this positive association was only present for the group with serum uric acid between 7.6 and 8.5 mg/dL in model 2 (OR, 0.62, 95% CI: 0.40–0.97, *p* < 0.05). Compared with the lowest uric acid level group, the OR for at least osteopenia was 0.78 (95% CI: 0.60–1.01, *p* = 0.06) for the group in which the serum uric acid level was between 6.6 and 7.5 mg/dL and 0.67 (0.38–1.17, *p* = 0.16) for the highest uric acid level group (*p* for the trend was <0.001) after adjustment for age, sex and BMI.

## 4. Discussion

This cross-sectional study focused on the association between the serum uric acid levels and BMD in Taiwanese older adults. Participants were classified by quartiles of the uric acid levels, which showed a nonlinear relationship. After adjusting for age, gender and BMI, the group with uric acid levels ≥ 6.6 mg/dL, which was traditionally defined as hyperuricemia, had a lower risk of at least osteopenia. Moreover, poor renal function might be a possible risk factor for osteoporosis; therefore, a subgroup analysis of CKD was performed. The results revealed no clear relationship between BMD and the population with eGFR < 60 mL/min/1.73 m^2^ (Supplementary Materials). People with hyperuricemia were more likely to have CKD, and it may inhibit serum uric acid’s bone protective effect of serum uric acid. A boxplot demonstrated that a higher BMD level was noted in the higher uric acid level group. The results from the multivariable linear regression model showed a consistent pattern. The serum uric acid level was positively correlated with both lumbar BMD and hip BMD. The β values indicated that the benefit from elevated serum uric acid was more in the lumbar spine. This finding could point the way forward for research into prevention policies for lumbar spine compression fractures in the elderly.

Recent studies have shown a negative relationship between hyperuricemia and the risk of low bone mass. A Korean nationwide cross-sectional study consisting of 173,209 participants > 40 years old adjusted their confounding factors, including BMI, age, gender, smoking, alcohol use, chronic disease history, nutrient intake and income. The results demonstrated that hyperuricemia was associated with a lower risk of osteoporosis [5]. The researchers collected data about a previous history of osteoporosis and other chronic diseases via interviews, so recall bias and diagnosis errors could not be ruled out. A study conducted in China investigated over 4000 participants aged 60–85 years old and found a positive relationship between serum uric acid and lumbar spine bone health [7]. The participants enrolled in that study shared similar ranges of age and ethnicity with our study, and the results were also consistent. In a study including a representative population in Taiwan, serum uric acid was found to have a dose-dependent osteoprotective role up to 8 mg/dL [6]. We studied an elderly population aged ≥ 60 years old and observed similar results. An analysis focusing on higher serum uric acid levels ≥ 6.6 mg/dL still showed a positive association with the BMD, which was more significant between 7.6 and 8.6 mg/dL after adjusting for confounding factors. However, the protective effect was not obvious in the group with levels ≥ 8.6 mg/dL. Clinically, urate-lowering therapy was considered for high-risk patients with serum uric acid concentrations > 9 mg/dL [12]. With no apparent benefit for bone protection and increased risk of a gout attack, asymptomatic hyperuricemia-related complications and urate-lowering therapy should be discussed with patients who have comorbidities.

Evidence from previous studies has shown the antioxidative effect of serum uric acid, which is the final oxidation product of the purine metabolic process in vivo [13]. As one of the major antioxidants and potent eliminators of oxygen radicals in the human body, serum uric acid has previously been shown to have the potential to inhibit osteoclastogenesis, which causes bone resorption [14,15]. Oxidative stress has been shown to stimulate the differentiation of osteoclastic cells and inhibit the differentiation of osteoblastic cells. Serum uric acid inhibits this process with an antioxidative effect, and it may contribute to bone formation [16]. Nevertheless, elevated serum uric acid and the crystallisation of monosodium urate accumulated in human joints may promote inflammatory cytokines and oxidative stress, leading to progressive bone loss [9]. Some studies have raised the concern that gout or hyperuricemia may be correlated with a fracture risk [17,18]. Due to the paradoxical role of serum uric acid reflected in its antioxidative properties and oxidative stress, more physiological evidence of the final effect on bone remodelling is needed.

In line with these findings, this study provided further evidence of a positive relationship between serum uric acid and BMD. This observation may also imply that the risk of low bone mass in elderly individuals will increase with lower levels of serum uric acid. There have been strong recommendations to treat patients with an indication for urate-lowering therapy to reach a serum uric acid goal of <6 mg/dL to optimise future outcomes [12]. Due to the increasing elderly population and the prevalence of osteoporosis, we need to take bone health into account. Clinical target values of serum uric acid may need to be revised. Higher uric acid levels in the elderly population may decrease the risk of at least osteopenia. For older adults who have lower uric acid levels, a BMD evaluation should be considered.

To our knowledge, this is the largest study in an elderly Taiwanese population to evaluate the association between serum uric acid and BMD. We collected both lumbar and hip BMD data so that we could diagnose low bone mass with a higher accuracy. The subgroup analysis of higher serum uric acid levels indicated that the protective effect of uric acid persisted until 8.6 mg/dL. The findings did not conflict with the current indications for urate-lowering therapy and also provided new reasons to reconsider the target goals of hyperuricemia treatment. Our findings add to the growing literature documenting the strong correlation between serum uric acid and BMD and suggest that serum uric acid screening may be important for the prevention of at least osteopenia. Extremely low serum uric acid levels could be considered to be a predictor for at least osteopenia and could help primary care physicians identify the risk of fragility fractures in older adults at an early stage.

Several potential limitations of this study should be considered. First, we used a cross-sectional design, which limited our ability to identify a causal correlation between serum uric acid and BMD in the elderly population. Second, there was no data on the history of gout attack episodes or fragility fractures from the medical records of the health evaluation centre. Relevant medication records such as urate-lowering therapy, osteoporosis medications, steroid, beta-blockers and hormone therapy were also lacking. This may have limited our ability to evaluate the effect of medication use on serum uric acid levels and hyperuricemia symptoms. Third, this study lacked a nutritional intake diary or serum albumin data, although we did adjust for the BMI in the logistic regression. We did not gain enough information on ethnicity, lifestyle risk factor and the physical activity assessment, which may influence the BMD. There might have been other possible confounding factors; thus, further studies with larger sample sizes are required to confirm the findings.

## 5. Conclusions

In conclusion, our results demonstrated that the serum uric acid level was positively correlated with BMD. The higher uric acid level in an elderly population may decrease the risk of at least osteopenia; thus, aggressive urate-lowering therapy should be carefully prescribed. Under extremely low serum uric acid levels, the benefit of urate-lowering therapy might not increase. For younger adults with a low risk of osteoporosis, hyperuricemic control is an important factor to avoid developing chronic disease. For older adults with lower uric acid levels, a BMD evaluation should be considered. Anti-hyperuricemic agents should be re-evaluated and adjusted in those patients whose serum uric acid level has already been under 6 mg/dL. To achieve a balance between osteoporosis and gout attacks, our results suggest that it would be important for primary care physicians to maintain proper uric acid levels in elderly individuals.

## Figures and Tables

**Table 1:** General characteristics of the participants.

Characteristics	All Participants				
	Q1	Q2	Q3	Q4	*p*
**Uric Acid (mg/dL)**	**<4.7**	**4.7–5.6**	**5.7–6.5**	**≥6.6**	
Age (years, %)					
60–69	401 (77.7)	465 (77.6)	435 (77.7)	425 (73.9)	<0.05
70–79	107 (20.7)	127 (21.2)	106 (18.9)	127 (22.1)	<0.05
≥80	8 (1.6)	7 (1.2)	19 (3.4)	23 (4)	<0.05
Sex (*n*, %)					
Men	138 (26.7)	266 (44.4)	339 (60.5)	420 (73.0)	<0.001
Women	378 (73.3)	333 (55.6)	221 (39.5)	155 (27.0)	<0.001
Body mass index (kg/m^2^, %)					
<24	353 (68.4)	343 (57.3)	249 (44.5)	211 (36.8)	<0.001
24–26.9	115 (22.3)	166 (27.7)	200 (35.7)	211 (36.8)	<0.001
≥27	48 (9.3)	90 (15)	111 (19.8)	152 (26.5)	<0.001
Comorbidities (*n*, %)					
Hypertension	145 (28.2)	210 (35.3)	195 (34.8)	219 (38.2)	<0.05
Diabetes mellitus	81 (24.5)	92 (24.5)	105 (29.7)	105 (28.5)	0.26
Hyperlipidaemia	317 (61.7)	416 (70)	372 (66.7)	427 (75)	<0.001
Thyroid disease	74 (14.5)	72 (12.1)	57 (10.3)	79 (13.9)	0.14
Chronic Kidney disease	11 (2.1)	24 (4)	31 (5.5)	102 (17.7)	<0.001
Bone Mineral Density (gm/cm^2^, SD)					
Lumbar	1.02 (0.18)	1.06 (0.2)	1.11 (0.2)	1.15 (0.2)	
Left hip	0.79 (0.13)	0.81 (0.13)	0.83 (0.14)	0.86 (0.16)	
T score (SD)					
Lumbar	−0.78 (1.47)	−0.45 (1.58)	−0.12 (1.61)	0.2 (1.63)	
Left hip	−1.18 (0.9)	−1.04 (0.99)	−1 (1.04)	−0.75 (1.16)	
Osteoporosis (n, %)	74 (14.5)	72 (12.1)	57 (10.3)	79 (13.9)	0.15
Osteopenia (n, %)	310 (71.1)	323 (64.3)	273 (58.3)	232 (42.2)	<0.001

Independent *t*-test or chi-square test. Significance at *p* < 0.05.

**Table 2:** Odds ratios (95% confidence interval) of the serum uric acid categories for at least osteopenia.

Serum Uric Acid Categories (mg/dL)	Q1 <4.7	Q2 4.7–5.6	Q3 5.7–6.5	Q4 ≥6.6
Crude OR (95% CI), *p* value	1	0.73 (0.56–0.97), <0.05	0.57 (0.43–0.75), <0.001	0.38 (0.29–0.50), <0.001
Model 1 OR (95% CI), *p* value	1	0.84 (0.63–1.12), 0.23	0.76 (0.57–1.02), 0.06	0.54 (0.41–0.73), <0.001
Model 2 OR (95% CI), *p* value	1	0.91 (0.68–1.22), 0.54	0.89 (0.66–1.20), 0.44	0.68 (0.50–0.92), <0.05

Logistic regression model. Significance at *p* < 0.05. Model 1 included age group (60–69 years, 70–79 years and ≥80 years). Model 2 included the same as model 1 plus sex and BMI group (<24 kg/m^2^, 24–26.9 kg/m^2^ and ≥27 kg/m^2^). OR, odds ratio; CI, confidence interval.

**Table 3:** Multivariable linear regression model showing an association between serum uric acid and bone mineral density (BMD).

	*β*	*p*-Value
	Serum uric acid (mg/dL)	
Lumbar BMD (g/cm^2^)	0.011	<0.05
Hip BMD (g/cm^2^)	0.005	<0.05

**Table 4:** Odds ratios (95% confidence interval) of the population with hyperuricemia for at least osteopenia.

Serum Uric Acid Categories (mg/dL)	<6.6	6.6–7.5	7.6–8.5	≥8.6
Crude odds ratios (95% CI) *p* value	1	0.55 (0.43–0.70) <0.001	0.46 (0.30–0.69) <0.001	0.44 (0.26–0.74) <0.001
Model 1 odds ratios (95% CI) *p* value	1	0.69 (0.54–0.89), <0.05	0.55 (0.36–0.84), <0.05	0.56 (0.32–0.96), <0.05
Model 2 odds ratios (95% CI) *p* value	1	0.78 (0.60–1.01), 0.06	0.62 (0.40–0.97), <0.05	0.67 (0.38–1.17), 0.16

Logistic regression model, *p* < 0.05.

## Data Availability

The data will be available upon reasonable request from the corresponding authors.

## Supplementary Materials

The following supporting information can be downloaded at: https://www.mdpi.com/article/10.3390/ijerph20043448/s1, Table S1: Subgroup analysis for the odds ratios (95% confidence interval) of serum uric acid categories for at least osteopenia according to the presense of chronic kidney disease.
