# Supplementary material for: Association between Serum Uric Acid Levels and Bone Mineral Density in Taiwanese Elderly Population
Source: Int J Environ Res Public Health. 2023 Feb 16;20(4):3448. doi: 10.3390/ijerph20043448 (PMC9961147; doi:10.3390/ijerph20043448)
Supplement: Supplementary file 1 [file ijerph-20-03448-s001.zip › ijerph-2127008-supplementary.pdf]

Table S1. Subgroup analysis for the odds ratios (95% confidence interval) of serum uric acid categories for at least osteopenia according to the presense of chronic kidney disease.

**Serum uric acid categories (mg/dl)**	**Q1 <4.7**	**Q2 4.7–5.6**	**Q3 5.7–6.5**	**Q4 ≥6.6**
eGFR≥60 ml/min/1.73m^2 OR, odds ratio; CI, confidence interval.	1	0.93 (0.69–1.26), 0.65	0.88 (0.65–1.19), 0.39	0.68 (0.50–0.93), <0.05
eGFR<60 ml/min/1.73m^2 OR, odds ratio; CI, confidence interval.	1	0.59 (0.10–3.36), 0.55	1.22 (0.22–6.74), 0.82	0.91 (0.20–4.16), 0.90

The model included age group (60–69 years, 70–79 years, ≥80 years), sex, BMI group (<24 kg/m^2, 24–26.9 kg/m^2, ≥27 kg/m^2)
